# Retraction: Zihan Xu, *et al.* Tanshinone IIA Pretreatment Renders Free Flaps against Hypoxic Injury through Activating Wnt Signaling and Upregulating Stem Cell-Related Biomarkers. *Int. J. Mol. Sci.* 2014, *15*, 18117–18130

**DOI:** 10.3390/ijms17050768

**Published:** 2016-05-20

**Authors:** International Journal of Molecular Sciences Editorial Office

**Affiliations:** MDPI AG, Klybeckstrasse 64, 4057 Basel, Switzerland; ijms@mdpi.com

We have been made aware that text, figures and experimental data reported in the title paper [1] are duplicated in another publication by Zihan Xu *et al.* [2]. At the time of submission, [1] had already been submitted to another journal and was under active consideration. The *International Journal of Molecular Sciences* is a member of the Committee on Publication Ethics and takes the responsibility to enforce strict ethical policies and standards very seriously. To ensure the addition of only high quality scientific works to the field of scholarly publication, paper [1] is retracted and shall be marked accordingly. We apologize to our readership that this went undetected until now.
